# Improvement of Prebiotic Properties and Resistant Starch Content of Corn Flour (*Zea mays* L.) Momala Gorontalo Using Physical, Chemical and Enzymatic Modification^*^

**DOI:** 10.21315/tlsr2023.34.2.13

**Published:** 2023-07-21

**Authors:** R. Haryo Bimo Setiarto, Muhammad Isra, Dimas Andrianto, Nunuk Widhyastuti, Masrukhin

**Affiliations:** 1 Research Centre for Applied Microbiology, National Research, and Innovation Agency (BRIN), Jalan Raya Jakarta-Bogor Km 46, Cibinong Science Centre, Cibinong, Bogor, 16911 West Java, Indonesia; 2 Department of Biology, Universitas Negeri Gorontalo, Kota Gorontalo, Gorontalo 96119, Indonesia; 3 Department of Biochemistry, IPB University, Bogor 16680 West Java, Indonesia; 4 Research Centre for Biosystematics and Evolution, National Research and Innovation Agency (BRIN), Jalan Raya Jakarta-Bogor Km 46, Cibinong Science Centre, Cibinong, Bogor, 16911 West Java, Indonesia

**Keywords:** Resistant Starch, Prebiotic Properties, Cornflour Momala Gorontalo, Starch Modification

## Abstract

Probiotics are a non-digestible food ingredient that promotes the growth of beneficial microorganisms in the intestines. One of the functional food ingredients, Momala corn flour, is a source of prebiotics with a resistant starch content of 4.42%. Thi s study aimed to improve the prebiotic properties and resistant starch content of modified corn flour (MCF) Momala Gorontalo by using physical, chemical, and enzymatic modification processes. The research methods include physical modification (heat moisture treatment, annealing, autoclaving-cooling cycling, microwave), chemical modification (acid hydrolysis), and enzymatic modification (debranching pullulanase). The results showed that the modified by heat moisture treatment (HMT) increased RS levels 1-fold, annealing modification (ANN) 8.9-fold, autoclaving-cooling one cycle modification (AC-1C) 2.9-fold, autoclaving-cooling two cycles modification (AC-2C) 2.0-fold, microwave modification (MW) 1.3-fold, acid hydrolysis (HA) modification 5.0-fold, and debranching pullulanase (DP) modification 3.8-fold compared with corn flour control without modification. The value of the prebiotic activity of MCF hydrolysed acid (HA) is 0.03, and debranching pullulanase (DP) is 0.02 against *Enteropathogenic Escherichia coli* (EPEC). The prebiotic effect value of MCF HA and DP were 0.76 and 0.60, respectively. The prebiotic index value of MCF HA and DP were 0.60 and 0.48, respectively. This study confirms that MCF HA and DP are good prebiotic candidates because they have resistant starch content, low starch digestibility, and resistance to simulated gastric fluid hydrolysis than unmodified corn flour.

**Highlights**

- The results showed that the modified by heat moisture treatment (HMT) increased RS levels 1-fold, annealing (ANN) modification 8.9-fold, autoclaving-cooling one cycle modification (AC-1C) 2.9-fold, autoclaving-cooling two cycles (AC-2C) modification 2.0-fold, microwave (MW) modification 1.3-fold, acid hydrolysis (HA) modification 5.0-fold, and debranching pullulanase (DP) modification 3.8-fold compared with corn flour control without modification.
- This study confirms that modified corn flour (MCF) acid hydrolysis (HA) and debranching pullulanase (DP) are good prebiotic candidates because they have resistant starch content, low starch digestibility, and resistance to simulated gastric fluid hydrolysis than unmodified corn flour.

## INTRODUCTION

Momala Gorontalo corn is one of Gorontalo’s local corn varieties from North Sulawesi, Indonesia, which has a different seed morphology from corn in general. Purple Momala corn seeds contains anthocyanins (Cono *et al*. 2021). Momala corn cultivation in Gorontalo Province has dramatically decreased due to the difficulty of obtaining seeds, the poor economic value of Momala corn, and the lack of public knowledge about the health benefits of Momala corn. Momala corn has a naturally occurring resistant starch type 1 (RS1), which is difficult to digest. Resistant starch type 1 (RS1) is starch found in foodstuffs physically trapped in plant cells and granule matrix, which is very susceptible to heat and physical, enzymatic treatment (Na *et al*. 2021). The physical characteristics of raw foods show that starch granules are composed of amylose and amylopectin molecules, causing natural starch susceptibility to enzymatic digestion (Barua *et al*. 2021). Therefore, there is a need for physical, chemical and enzymatic modifications to regulate the digestive properties of starch so that it is more difficult to digest by human digestive enzymes and has prebiotic properties to be utilised by probiotic bacteria in the colon.

Prebiotics are food ingredients that are difficult to digest by acid in the stomach and digestive enzymes in the small intestine. So, it can reach the large intestine to increase the growth of probiotic bacteria in the colon (Zailani *et al*. 2022). Prebiotics has been widely commercialised in supplements and functional food, such as oligosaccharides, raffinose, and inulin (Chang *et al*. 2020). Another prebiotic has the potential as a source of prebiotics, namely resistant starch. Tu *et al*. (2021) reported that prebiotics is food ingredients that are selectively able to stimulate the growth of probiotic bacteria in the colon. Resistant starch is the starch that cannot be digested by digestive enzymes, resistant to gastric acid, so it can reach the large intestine to be fermented by probiotic bacteria.

Physical modification methods (autoclaving-cooling, HMT, annealing, microwave), chemical modification (acid hydrolysis) and enzymatically with pullulanase debranching were able to increase the levels of resistant starch through gelatinization and retrogradation to form double-helical bonds (Zailani *et al*. 2022). The mechanism of action of the pullulanase enzyme by breaking branching α-1,6 glycosidic bonds can change the structure and amount of short-chain amylose so that it can increase RS levels (Tu *et al*. 2021). However, several studies using physical, chemical, and enzymatic modifications in food showed different prebiotic properties and levels of resistant starch. Hence, this study aimed to investigate the improvement of resistant starch content and prebiotic properties of cornflour (*Zea mays*, L.) Momala Gorontalo by using physical, chemical and enzymatic modification.

## MATERIALS AND METHODS

### Materials

The main ingredient of this research was Gorontalo momala corn, which was obtained from Mulyonegoro Village, Pulubala District, Gorontalo Province, Indonesia. Coordinate point 0***°***37′52.2″ N, 122***°***43′34.9″ E. Probiotic bacteria *Lactobacillus plantarum* IIA-1A5 and *Enteropathogenic Escherichia coli* (EPEC) culture from livestock technology laboratory, Faculty of Animal Science, IPB University, Bogor, Indonesia.

### Production of Momala Gorontalo Corn Flour

The Momala Gorontalo corn used was a 5-month-old corn plant. Momala Gorontalo corn flour is processed corn, using whole corn kernels that have been peeled from the corn cob. Then ground using a disc mill FFC-15 Shangdao (China), water immersion was carried out to separate the impurities. Grits can be obtained by finer milling, and re-drying was carried out by sifting with 80 mesh to get Momala corn flour.

### Modification Momala Corn Flour (MCF) by Acid Hydrolysis

Modified acid hydrolysis (HA) was used to increase the levels of resistant starch, according to methods used by Sudheesh *et al*. (2020). Amount 250 g corn flour was added with 250 mL HCl (Merck, Germany) in a 2.2 N ratio (1:1), then incubated in a 35***°***C water bath incubator (Memmert, Germany) for 2 h. Furthermore, 1 M NaOH (Merck, Germany) was added to pH 6.0, then washed twice with aquadest. Modified corn flour (MCF) samples were incubated at room temperature for 60 min and stored in a cooler room for 24 h at 4***°***C. Oven (Isuzu Seisakusho Co., LTD, Japan) for 24 h at 60***°***C to form dry flour pieces in a blender and sieved (mesh 80).

### Modification MCF by Annealing

Modification by annealing (ANN) to increase resistant starch content according to methods used by Chung *et al*. (2019). Corn flour 100 g was added with 500 mL of distilled water in a ratio (1:5) 30% w/v, then incubated in a 50***°***C water bath incubator (Memmert, Germany) for 24 h. MCF samples were incubated at room temperature for 60 min and stored in a cooler room for 24 h at 4***°***C. The MCF sample was dried in an oven (Isuzu Seisakusho Co., LTD, Japan) for 24 h at 60***°***C to form dry flour chips, blended and sieved (mesh 80).

### Modification of MCF by Autoclaving-cooling

Autoclaving-cooling (AC) modification improved the resistant starch, according to methods used by Putra (2020). Corn flour 100 g was added with 300 mL of distilled water in a ratio (1:3) of 20% w/v. Moreover, it was autoclaved (121***°***C, 15 min) by autoclave (Hirayama Manufacturing Corp., Japan) and then refrigerated in a cooler room (4***°***C, 24 h). This modification was repeated for a 2-cycle autoclaving-cooling improvement. The MCF sample was dried (24 h, 60***°***C) in an oven (Isuzu Seisakusho Co., Ltd, Japan) to form flour pieces, blended and sieved (80 mesh).

### Modification of Momala Corn Flour (MCF) by Heat Moisture Treatment

Modification of heat moisture treatment (HMT) was modified to increase resistant starch content, according to methods used by Chang *et al*. (2020) and Chung *et al*. (2019). Amount 100 g corn flour laid out on a metal baking sheet, then added aquadest until it reached a moisture content of 20%. Next, the corn flour was dried in the oven (Isuzu Seisakusho Co., LTD, Japan) (120***°***C, 3 h). The flour chips were incubated for 60 min and stored in a cooler room (4***°***C, 24 h). The MCF sample was dried in the oven (Isuzu Seisakusho Co., LTD, Japan) (60***°***C, 24 h), forming dry flour pieces, blended, and sieved (mesh 80).

### Modification of Momala Corn Flour (MCF) by Debranching Pullulanase

Modification of debranching pullulanase (DP) to increase resistant starch content according to methods used by Tu *et al*. (2021) and Huang *et al*. (2015). Amount 100 g corn flour was added with 300 mL of distilled water (1:3) 20% w/v, then incubated in a 70***°***C water bath for 5 min. The autoclaving process uses an autoclave (Hirayama Manufacturing Corp) (121***°***C, 15 min). Then, corn flour was refrigerated in a cooler room (4***°***C, 24 h). Furthermore, it was incubated with a 50***°***C water bath, adding 100 mL of 0.1 M acetate buffer pH 5.2, and 2.5 mL of pullulanase enzyme SIGMA (E2412-250ML) (10.4U/g flour). Moreover, it was incubated with a shaker incubator (Bio-Shaker BR-300LF) (50***°***C,110 rpm, 24 h). The pullulanase enzyme inactivation was carried out by autoclaving-cooling for one cycle. The MCF sample was dried using an oven (Isuzu Seisakusho Co. Ltd., Japan) (24 h, 60***°***C) to form dry flour chips, blended and sieved (mesh 80).

### Modification of MCF by Microwave

Microwave (MW) modification to improve resistant starch content according to methods used by Barua *et al*. (2021). Amount 100 g corn flour was added with 500 mL (1:5) 30% w/v aquadest. Then, it was exposed to heat using a microwave (SHARP) input 0.7 kW 3.6 A, output 399 Watt for 13 min. MCF samples were incubated for 60 min and stored in a cooler room (4***°***C, 24 h). The MCF sample was dried using an oven (Isuzu Seisakusho Co., Ltd, Japan) (60***°***C, 24 h), forming dry flour pieces, blended and sieved (mesh 80).

### Analysis Microstructure of MCF by Scanning Electron Microscope

Scanning electron microscope (SEM) analysis was performed using the modified method of Zavarezel *et al*. (2021) to determine the microstructure and particle size of MCF. A sputter coater (Hitachi E 102 MC1000 Ion Sputter) having a 20 nm thick gold layer with a conductive double-sided carbon adhesive tape was used to cover the MCF powder. Surface and cross-sectional visualisation of MCF starch granules was carried out by placing the film on an aluminum sheet recorded with double-sided tape and then covered with a 20 nm–30 nm thick gold layer. An accelerating voltage of 20.0 kV was used to analyse all MCF starch granule samples. SEM Hitachi S 2400 recorded and analysed MCF starch granules. Images of MCF starch granules were visualised up to 1.000 × sizes 1280 *×* 960 pixels using Image J software.

### Analysis Total Starch Content of MCF

Total starch content of MCF using the method developed by Dubois *et al*. (1956). Amount 0.5 mL of the sample was put into a test tube, then 0.5 mL of 5% phenol was added and homogenised using a vortex. H_2_SO_4_ solution concentrated 2.5 mL was added rapidly into the test tube, resulting in an exothermic reaction that produced heat. The sample solution was then allowed to stand for 10 min at room temperature, vortexed, and stand for another 20 min at room temperature. The absorbance value was measured by a spectrophotometer UV-Vis (Shimadzu, Japan) at a wavelength of 490 nm. Glucose level (μg/mL) was determined using a standard curve. The total sugar content (% dry weight) was obtained from the standard curve, while the total starch content (% dry weight) of MCF was calculated by multiplying the total sugar content by 0.9.


$$\begin{array}{l} \text{Total sugar content }(\%\;\text{dw})=\frac{\text{Glucose level }(\text{mg}/\text{mL})}{\text{Sample weight }(\text{mg})}\times \text{volume total reaction }(\text{mL})\times \text{Dilution Factor}\times 100\%\\ \text{Total starch content }(\%\;\text{dw})=\text{Total sugar content }(\%\;\text{dw})\times 0.9 \end{array}$$

where 0.9 is used to represent experimental factors for the conversion of monosaccharides to polysaccharides.

### Analysis of Amylose and Amylopectin Content of MCF

Analysis of amylose content was carried out followed the IRRI (1979) method. Amount 0.1 g of MCF sample were added with 1 mL of 95% ethanol, and 9 mL of 1 N NaOH solution put into a 100 mL volumetric flask. The flask was heated in a water bath (10 min, 95***°***C). After cooling, the MCF gel solution was added with distilled water to the mark and homogenised using a vortex. Amount 5 mL of MCF gel solution was pipetted and transferred to a test tube. Then, it was added with 1 mL of 1 N acetic acid solution, 2 mL of iodine solution, 5 mL of aquadest into a measuring flask. The sample solution was allowed to stand for 20 min at room temperature before measuring its absorbance with a spectrophotometer UV-Vis (Shimadzu, Japan) at a wavelength of 625 nm. Amylose content (%) was determined using the standard curve equation of amylose solution.


$$\begin{array}{l} \text{Amylose content }(\%\;\text{dw})=\text{Amylose content }(\text{mg}/\text{mL})\;\text{Sample weight }(\text{mg})\times \text{total reaction volume }(\text{mL})\times \text{DF}\times 100\%\\ \text{Amylopectin content }(\%\;\text{dw})=\text{Total starch content }(\%\;\text{dw})-\text{Amylose content }(\%\;\text{dw}) \end{array}$$

### Analysis of Starch Digestibility *in vitro* of MCF

*In vitro* starch digestibility of MCF was analysed by measuring the maltose level as the product of hydrolysis MCF by using α-amylase from *Aspergillus oryzae* (Sigma 10065-10G) 100 U compared to starch solution. This analysis was performed by referring to a method from Otemuyiwa and Aina (2021). Sample absorbance and blank solution were determined by Spectrophotometer UV-Vis (Shimadzu UV-1800, Tokyo, Japan) at 520 nm. Blank samples and blank pure starch are sample and pure starch solutions without treatment of α-amylase enzyme from *Aspergillus oryzae*. Blank samples and blank pure starch were used as negative controls to detect maltose levels in samples and pure starch. Starch digestibility was calculated by comparing the maltose content from the sample with the pure starch. Maltose results from starch hydrolysis by the α-amylase enzyme from *Aspergillus oryzae*. In this study, the calculation of the starch digestibility (%) was shown in the following formula:


$$\text{Starch digestibility }(\%)=\frac{\text{Maltose content of sample}-\text{maltose content of blank sample}}{\text{Maltose content of pure starch}-\text{maltose content of blank pure starch}}\times 100\%$$

### Analysis of Starch Composition of MCF

The digestible starch composition analysis of MCF was conducted in this study by following Zailani *et al*. (2022) method. MCF was extracted first to obtain pure corn starch for VRDS, RDS, SDS and RS analysis. There are four types of MCF starch compositions based on their digestibility times. The first type is called very rapidly digestible starch (VRDS), which is expressed as the amount of digested starch in the first minute by porcine pancreatin and amyloglucosidase 210 U as explained in the Sigma Cat. No. P7545 and No. A7095, respectively. The second type is called the rapidly digestible starch (RDS) which is the amount of digested starch expelled between 1 min and 20 min. The third type is the slowly digestible starch (SDS) which is expressed as the amount of digested starch between 20 min and 120 min. Finally, the resistant starch (RS) is described as non-digestible starch after 120 min of analysis. The level of glucose within digested supernatant was spectrophotometer UV-Vis (Shimadzu UV-1800, Tokyo, Japan) at 540 nm. The following equations were used in the calculations.


$$\begin{array}{l} \text{VRDS }(\%)=\frac{\text{G}1\times 0.9\times \text{F}}{\text{W}}\times 100\\ \text{RDS }(\%)=\frac{(\text{G}20\text{-G}1)\times 0.9\times \text{F}}{\text{W}}\times 100\\ \text{SDS }(\%)=\frac{(\text{G}120\text{-G}20)\times 0.9\times \text{F}}{\text{W}}\times 100\\ \text{RS }(\%)=100-\text{VRDS}-\text{RDS}-\text{SDS} \end{array}$$

where G1 = The absorbance of glucose after 1 min incubation; G20 = The absorbance of glucose after 20 min incubation; G120 = The absorbance of glucose after 120 min incubation; F = 100/absorbance is the dividing value for absorbance to get the percentage of flour that has very rapidly digestible starch (VRDS), rapidly digestible starch (RDS), and slowly digestible starch (SDS), and resistant starch (RS) divided by sample weight (W); W = sample weight. (0.9 is used to represent an experimental factor to convert monosaccharaides into polysaccharides)

### Analysis Resistance of MCF against Simulated Gastric Fluid

Resistance of MCF to simulated gastric fluid using a method developed by Otemuyiwa and Aina (2021). MCF was prepared by dissolving modified taro starch into sterile distilled water (1% w/v). Gastric acid simulated is a hydrochloric acid buffer which per gram/liter contains: NaCl (8 g/L); KCl (0.2 g/L); Na_2_HPO_4_.2H_2_O (8.25 g/L); NaH_2_PO4 (14.35 g/L); CaCl_2_.2H_2_O (0.1 g/L); MgCl_2_.6H_2_O (0.18 g/L). The hydrochloric acid buffer was conditioned at pH 2 using HCl 5 M. A total of 5 mL of HCl buffer for each pH treatment was poured to 5 mL of solution, and then incubated in the water bath at 37***°***C for 6 h. They were analysed at 0 h, 0.5 h, 2 h, 4 h and 6 h. The levels of reducing sugars were measured using the DNS method (Chang *et al*. 2020) and the total sugar content was determined by the sulfuric acid-phenol method (Oh *et al*. 2022). The percentage (%) of hydrolysis MCF is calculated using equation according to Lu *et al*. (2021).


$$\begin{array}{l} \text{Hydrolysis }(\%)=\frac{\text{reducing sugar content}}{\text{total sugar content}}\times 100\\ \text{Resistance of MCF }(\%)=100\%-\text{The percentage }(\%)\;\text{of hydrolysis MCF} \end{array}$$

### Analysis Prebiotic Activity against *Enteropathogenic Escherichia coli* (EPEC) for MCF

MCF viability and prebiotic activity assays were carried out using a modified procedure of Putra (2020) and Tu *et al*. (2021). The examination of prebiotic activity was conducted by adding 2% (v/v) of *L. plantarum* IIA-1A5 culture into m-MSRB with 2.5% (w/v) of glucose or 2.5% (w/v) of MCF (HA, annealing, autoclaving-cooling 1 cycle, autoclaving-cooling 2 cycles, heat moisture treatment, debranching pullulanase, microwave-cooling and control). At 0 h and 24 h of incubation times, the samples were calculated in the Methicillin-resistant *Staphylococcus aureus* (MRSA) medium. The examination was also conducted towards diarrhea-causal-bacteria, *Enteropathogenic Escherichia coli* (EPEC). The EPEC culture of 2% (v/v) was added into different Erlenmeyer containing m-TSB (Tryptone Soy Broth) 2.5% (w/v) of glucose or 2.5% (w/v) MCF (HA, annealing, autoclaving-cooling 1 cycle, autoclaving-cooling 2 cycles, heat moisture treatment, debranching pullulanase, microwave-cooling, and control). The cultures were incubated at 37°C and calculated in the TSA medium after 0 h and 24 h of incubation times. The value of prebiotic activity was analysed by determining a positive value (+) indicating the growth of probiotic bacteria *L. plantarum* IIA-1A5 while a negative value (−) indicating the occurrence of EPEC growth resulting from the equation:


$$\begin{array}{ll} \text{Prebiotic activity value } & =\left\{\frac{N\;\text{log}\left(\frac{\text{cfu}}{\text{mL}}\right)\text{MCF }t_{1}-N\;\text{log}\left(\frac{\text{cfu}}{\text{mL}}\right)\text{Glucose }t_{0}}{N\;\text{log}\left(\frac{\text{cfu}}{\text{mL}}\right)\text{MCF }t_{1}-N\;\text{log}\left(\frac{\text{cfu}}{\text{mL}}\right)\text{Glucose }t_{0}}\right\}\\ & -\left\{\frac{E\;\text{log}\left(\frac{\text{cfu}}{\text{mL}}\right)\text{taro starch }t_{1}-E\;\text{log}\left(\frac{\text{cfu}}{\text{mL}}\right)\text{taro starch }t_{0}}{E\;\text{log}\left(\frac{\text{cfu}}{\text{mL}}\right)\text{Glucose }t_{1}-E\;\text{log}\left(\frac{\text{cfu}}{\text{mL}}\right)\text{Glucose }t_{0}}\right\} \end{array}$$

where N = number of *L. plantarum* SU-LS 36 (log cfu/mL); *t*_0_ = start of incubation time (0 h); E = number of *EPEC* (log cfu/mL); and *t*_1_ = end of incubation time (24 h).

### Analysis Prebiotic Effect and Index of MCF

*L. plantarum* IIA-1A5 was cultivated in MRS broth (Oxoid Ltd., Hampshire, England) (1:100) (v/v) and incubated (24 h, 37***°***C). *L. plantarum* IIA-1A5 cell biomass was harvested using a high-speed centrifuge 6500 (Kubota, Tokyo, Japan) (5000 *g*, 20 min, 4***°***C) until it reached concentration of *L. plantarum* IIA-1A5 cell biomass (10^7^ CFU/g). The analysis of prebiotic effect and prebiotic index was conducted by observing the change in the number of *L. plantarum* IIA-1A5 colonies on m-MSRB medium and m-MSRB medium with 2.5% taro starch (native, AC-1C, AC-2C, AC-3C, annealing and HMT). They were determined using the methods by Janggu *et al*. (2021). After the 24 h at 37°C incubation process, the probiotic cell cultures were enumerated in the MRSA medium. The same procedure was carried out using media with and without a carbon source as a comparison. The calculations were finished using this following equation:


$$\begin{array}{l} \text{Prebiotic effect}=\text{log}\left(\frac{\text{CFU}}{\text{mL}}\right)2.5\%\text{MCF}-\text{log}\left(\frac{\text{CFU}}{\text{mL}}\right)\text{m}-\text{MRSB}\\ \text{Prebiotic index}=\frac{\text{log}\left(\frac{\text{CFU}}{\text{mL}}\right)2.5\%\text{MCF}-\text{log}\left(\frac{\text{CFU}}{\text{mL}}\right)\text{mMRSB}}{\text{MCF weight}} \end{array}$$

### Statistical Data Analysis

ANOVA analysis was carried out to determine whether there were differences in the variables tested in this case: starch digestibility *in vitro*, the profile of starch composition of MCF, the prebiotic effect, index and activity of MCF. The test was carried out at the 95% significance level (α = 5%) with Duncan’s further test (Multiple Range Test). This variance analysis used SPSS (Statistical Package for Social Science) 26.00 software.

## RESULTS AND DISCUSSION

### Scanning Electron Microscope Analysis

**Figure 1:**
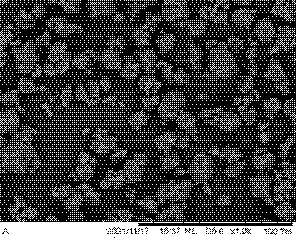

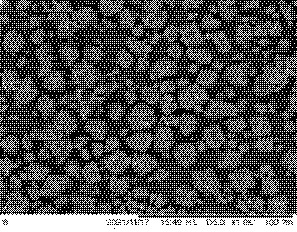

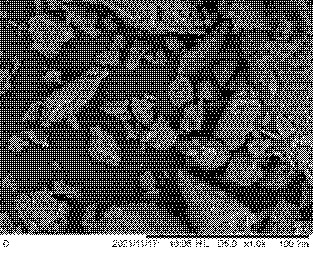

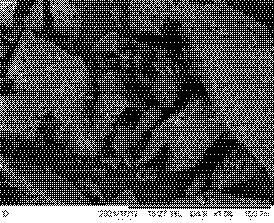

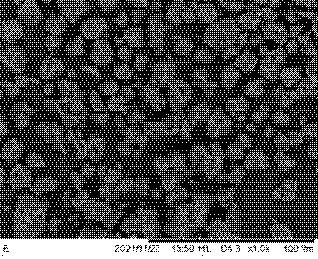

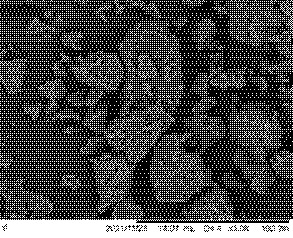

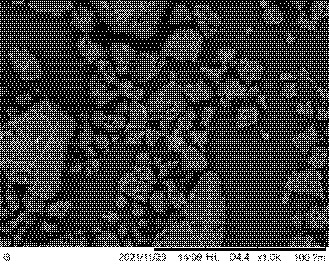

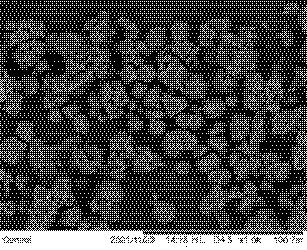
SEM of microstructure analysis of flour after modification. (A) acid hydrolysis; (B) annealing; (C) autoclaving-cooling 1 cycle; (D) autoclaving-cooling 2 cycles; (E) heat moisture treatment; (F) debranching pullulanase; (G) microwave-cooling; and control.

In this study, the microstructure of the MCF granules was observed using SEM with 1000× magnification. MCF tested as many as eight samples based on the treatment of physical, chemical, and enzymatic modifications. The morphology of the corn starch granule structure is quite diverse, namely the surface of the grain of various lumps such as round with rough and smooth surfaces and irregular polygons (Fig. 1). The microstructure of the control corn starch granules was used as a comparison for each treatment. It was observed that the control cornstarch granules showed a mixed structure of various sizes, and the starch granules were domes, round, polygon, broken and irregular.

Modifications of acid hydrolysis, microwave-cooling, debranching pullulanase, and autoclaving-cooling one cycle and two cycles have undergone complete gelatinisation. Acid hydrolysis can break glycosidic bonds, causing amylose chain shortening and the molecular weight of amylose to be lower, causing a decrease in flour’s stability into a paste during the gelatinisation process. It was resulted in the formation of a starch gel (paste) to form an irregular and coarse lump structure. The starch granules have broken due to the gelatinisation and retrogradation processes, including crystallisation. Zeng *et al*. (2019) reported that crystallisation on kudzu starch occurs because of gelatinisation and retrogradation. Gelatinisation is caused by different humidity and thermal energy.

### Total Sugar Content of MCF

**Figure 2:**
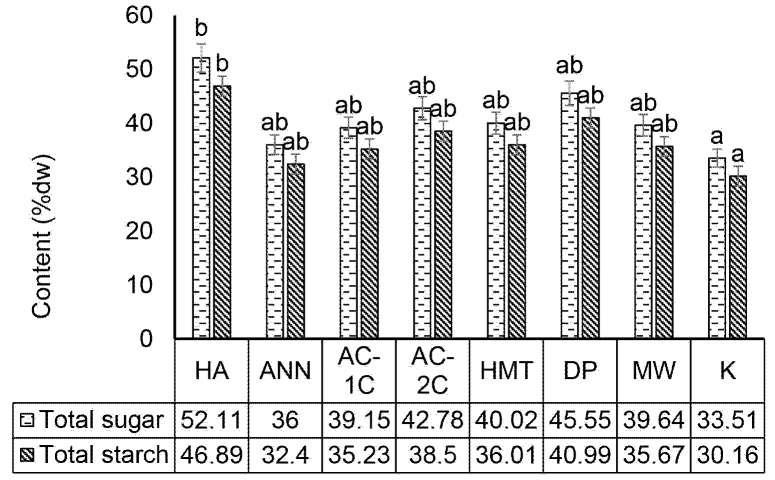
Effect of physical, chemical and enzymatic modifications on the total sugar and starch of modified corn flour. HA (acid hydrolysis), ANN (annealing), AC-1C (autoclaving-cooling 1 cycle), AC-2C (autoclaving-cooling 2 cycles), HMT (heat moisture treatment), DP (debranching pullulanase), MW (microwave-cooling) and K (control). The same letter in the bar chart shows a value that is not significantly different with a significance level of 95% (α = 5%), after statistical testing with Duncan on SPSS 26.0.

Chemical modification with acid hydrolysis had a significant effect (*p* < 0.05) in increasing the total sugar content when compared to other modifications and control without modification. The complex structure of starch has been hydrolysed into simpler compounds through the retrogradation of long-chain amylose into short chains, causing the total starch and total sugar levels to increase (Zhong *et al*. 2017). Modified acid hydrolysis had the highest total sugar content significantly (*p* < 0.05) at 52.11% dry weight (dw) (Fig. 2). The interaction between acid and corn starch increases the amount of short-chain amylose fraction with low molecular weight.

Total sugar is a carbohydrate compound that is either a monosaccharide or disaccharide (glucose, galactose, fructose, sucrose) that gives a sweet taste (Mutlu *et al*. 2018). Studies on total sugar content with modified acid hydrolysis have been carried out with other foodstuffs with varying increases in red beans at 12.16% (dw), sago at 13.84% (dw) and corn at 20.41% (dw) (Ye *et al*. 2018; Ji & Yu 2018; Zailani *et al*. 2022). Annealing modification has a total sugar of 36% (dw) as shown in Fig. 2. Annealing modification was carried out by using large concentrations of water (more than 40%) and heat treatment at temperatures below the gelatinisation temperature of starch, causing the viscosity of the paste to increase, which tends to undergo a retrogradation process (Deka & Sit 2016). Corn starch modification by annealing can reduce amylopectin levels and increases short-chain amylose levels. Annealing treatment results in partial gelatinisation which can provide linearisation and debranching effect of the alfa-1,6 glycosidic bond from amylopectin. Short-chain amylose is a raw material to improve resistant starch levels in corn flour by using retrogradation and annealing treatment.

### Total Starch Content of MCF

Chemical modification with acid hydrolysis had a significant effect (*p* < 0.05) in increasing the total starch content when compared to other modifications and control without modification. Changes in the total starch content are caused by starch hydrolysis which can break the starch chain into simpler and more water-soluble hydrolysates (Ji & Yu 2018). The physical, chemical, and enzymatic modification resulted in total starch which was significantly different (*p* < 0.05) from the control, which was 30.16% dry weight (Fig. 2). Changes during the heating-cooling process and hydrolysis of starch complex structures become simpler, thereby increasing the total starch content. Total starch content is strongly influenced by amylose and amylopectin content which are very susceptible to physical changes such as heating and cooling (Zailani *et al*. 2022).

Chemical modification by acid hydrolysis had the highest total starch content 46.89% (dw). The interaction between acid and corn starch forms a linear amylose chain. Huang *et al*. (2015) reported that acid hydrolysis using HCl could increase the total starch content of potato 17% (dw) and sorghum 15% (dw). The acid will hydrolyse starch into glucose (sugar). The acid hydrolysis process in starch converts starch molecules into monomers.

### Amylopectin and Amylose Levels of Modified Corn Flour

**Figure 3:**
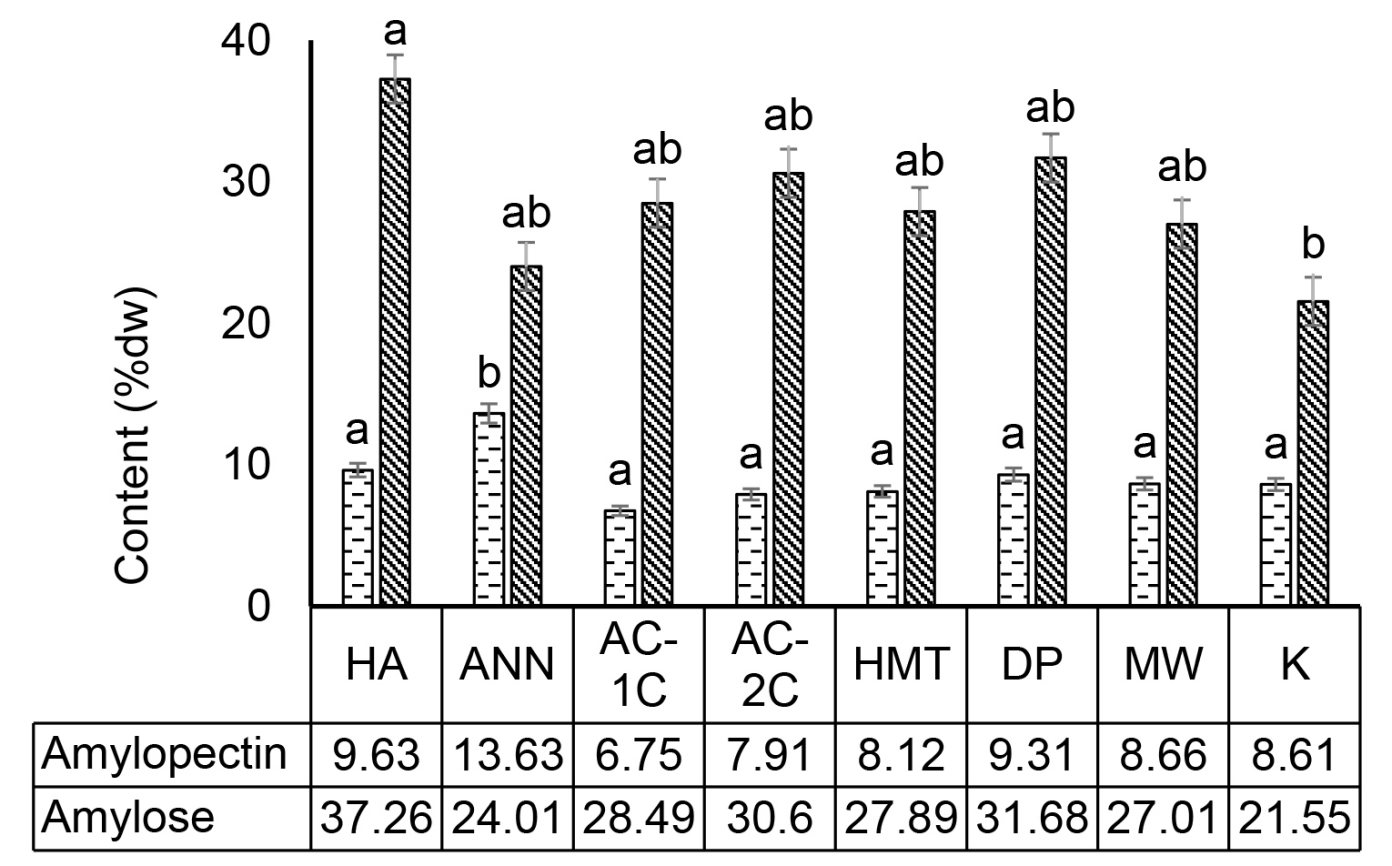
Effect of physical, chemical, and enzymatic modification on the amylopectin and amylose content of modified corn flour. HA (acid hydrolysis), ANN (annealing), AC-1C (autoclaving-cooling 1 cycle), AC-2C (autoclaving-cooling 2 cycles), HMT (heat moisture treatment), DP (debranching pullulanase), MW (microwave-cooling), cooling) and K (control). The same letter in the bar chart shows a value that is not significantly different with a significance level of 95% (α = 5%), after statistical testing with Duncan on SPSS 26.0.

Chemical modification by acid hydrolysis and physical modification of annealing significantly (*p* < 0.05) increased amylose content and decreased amylopectin content in MCF. Modified acid hydrolysis (HA) significantly (*p* < 0.05) increased amylose content by 37.26% dry weight in MCF when compared to control (K) which was 21.55% (dw). The increase in amylose content caused a significant decrease in amylopectin levels (*p* < 0.05) in acid hydrolysis MCF and was followed by other modifications significantly (*p* < 0.05) (Fig. 3).

Acid hydrolysis breaks amylopectin bonds randomly and produces smaller short chain amylose causing higher amylose content than control. It has been reported in maize starch that the amylopectin content of 11.02% (dw) and amylose of 20.32% (dw) were induced by stearic acid and produced a smaller short chain amylose structure (Mapengo *et al*. 2021). Modification by autoclaving-cooling had a significant effect (*p* < 0.05) in increasing amylose levels and reducing amylopectin levels in MCF. The more autoclaving-cooling cycles carried out, the more significant impact occurred on increasing amylose levels and decreasing amylopectin levels. Modification of 1 cycle autoclaving-cooling (AC-1C) and 2 cycles (AC-2C) significantly (*p* < 0.05) increased amylose content in MCF. The amylose content of MCF AC-1C increased by 28.49% (dw) and AC-2C 30.06% (dw) when compared to the control (K) which was 21.55% (dw). The decrease in MCF AC-1C amylopectin levels by 6.75% (dw) and AC-1C 7.91% (dw) occurred significantly (*p* < 0.05) when compared to control (K) 8.61% (dw) (Fig. 3).

The increase in amylose in each autoclaving-cooling modification was due to the hydrolysis process using a temperature of 121***°***C. There was a change in the structure of branched amylopectin into short-chain amylose fractions, breaking off a small portion of glycosidic bonds in branching α-1,6 made the changes in amylopectin branched structure become linear short chain amylose in rice and corn starch (Thuengtung *et al*. 2019). Linearisation of amylopectin components into short-chain amylose during autoclave heating causes an increase in amylose content and a decrease in amylopectin content (Moongngarm 2013).

### Digestibility *in vitro* of MCF

**Figure 4:**
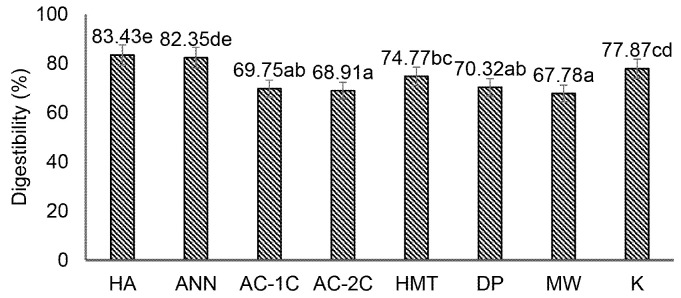
Effect of physical, chemical, and enzymatic modifications on the digestibility of modified corn flour. HA (acid hydrolysis), ANN (annealing), AC-1C (1 cycle autoclaving-cooling), AC-2C (autoclaving-cooling 2 cycles), HMT (heat moisture treatment), DP (debranching pullulanase), MW (microwave) and K (control). The same letters in the bar chart show values that are not significantly different with a significance level of 95% (α = 5%), after statistical testing with Duncan on SPSS 26.0.

Physical modification (HMT, annealing, autoclaving-cooling 1 cycle and 2 cycles, microwave), chemical modification by acid hydrolysis, enzymatic modification by debranching pullulanase had different digestibility changes. Modifications of debranching pullulanase (DP), autoclaving-cooling 1 cycle (AC-1C), 2 cycles (AC-2C), and microwave (MW) significantly (*p* < 0.05) in reducing the digestibility of corn flour when compared with control (K) which is 77.87 (Fig. 4).

The increase in digestibility in the acid hydrolysis (HA) and annealing treatment was because, during starch hydrolysis, the two modifications had high sugar content, which caused the digestibility to increase. The acid hydrolysis and annealing processes are only partially gelatinised so that the double helix structure is not wholly formed (Ye *et al*. 2018). The random hydrolysis of starch causes the formation of short-chain amylose, oligosaccharides, maltose, maltotriose and glucose with lower molecular weight so that it is easier to digest and causes an increase in the glycemic index (Piecyk & Domian 2021).

Modifications (HMT, AC-1C, AC-2C, DP, MW) with high temperature treatment caused the number of short amylose chains to form crystallisation (Larder *et al*. 2019). Crystallisation makes the bonds between amylose-amylose and amylose-amylopectin denser, resulting in retrograded hydrogen bonds that are more solid and difficult to digest (Lu *et al*. 2021). Retrograded MCF is difficult to digest, one of the reasons is that the enzyme α-amylase is difficult to recognise double helix bonds in resistant starch due to gelatinisation and retrogradation processes.

### Starch Composition of Modified Corn Flour

Starch is classified into several categories based on its digestibility: fast-digesting starch, fast-digesting starch, slow-digesting starch, and resistant starch. Starch classification based on starch digestibility was consistent with starch digestibility as described by Englyst *et al*. (1992) that very rapidly resistant starch (VRDS) is starch hydrolysed within 1 min. Rapidly digestible starch (RDS) is hydrolysed starch within 20 min of incubation. Slowly digestible starch (SDS) is starch that is digested over a period between 20 min and 120 min. The fourth category is resistant starch (RS) because starch is not hydrolysed for more than 120 min.

**Figure 5:**
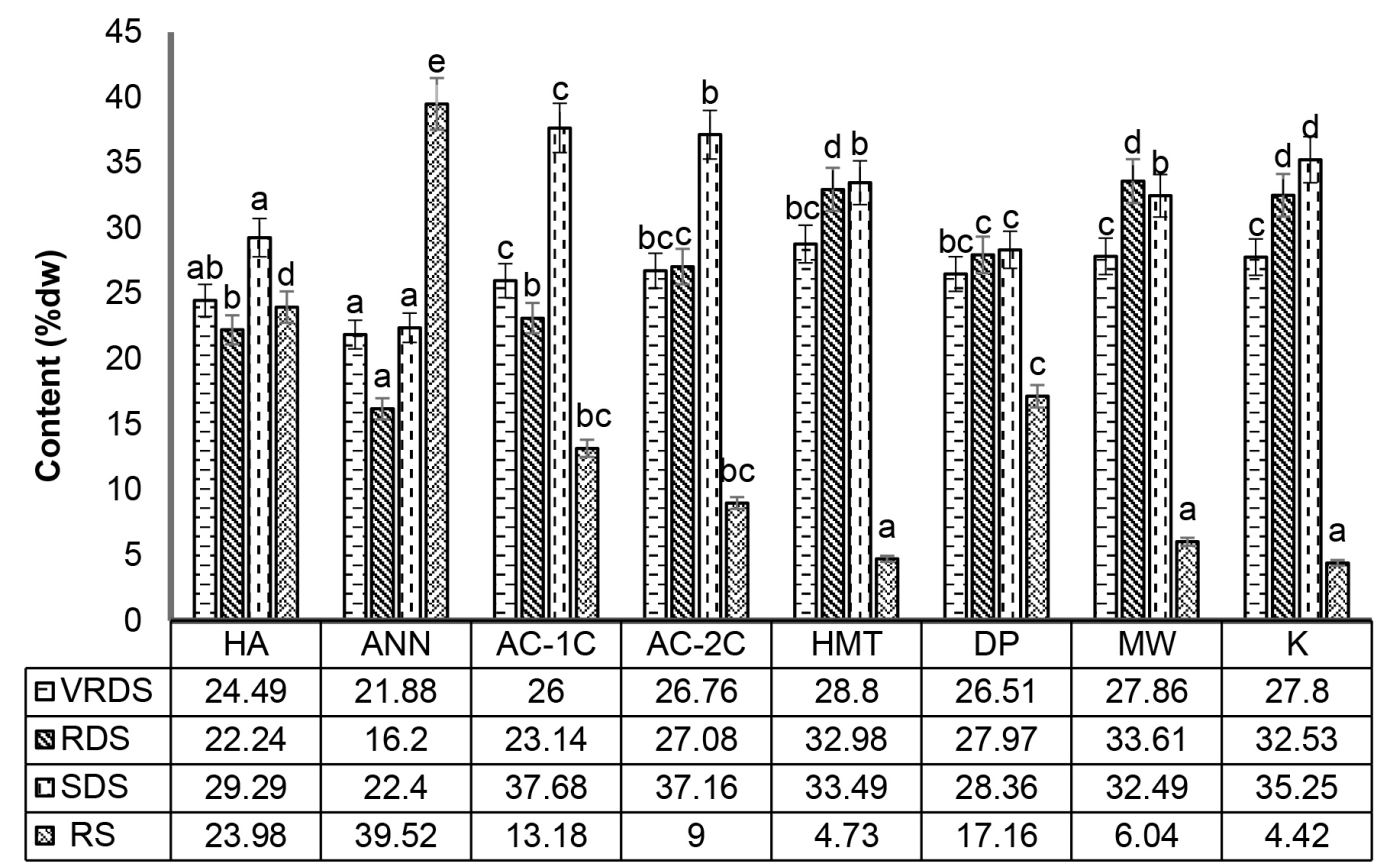
Effect of physical, chemical, and enzymatic modification on very rapidly digestible starch (VRDS), rapidly digestible starch (RDS), slowly digestible starch (SDS), and resistant starch (RS) of modified corn flour. HA (acid hydrolysis), ANN (annealing), AC-1C (1 cycle autoclaving-cooling), AC-2C (autoclaving-cooling 2 cycles), HMT (heat moisture treatment), DP (debranching pullulanase), MW (microwave) and K (control). The same letters in the bar chart show values that are not significantly different with a significance level of 95% (*α* = 5%), after statistical testing with Duncan on SPSS 26.0.

Modified HA and physical modification with annealing (ANN) significantly (*p* < 0.05) decreased the levels of VRSD and RDS of cornstarch when compared to K and increased levels of SDS and RS. There was a change in the structure of VRDS and RDS to SDS and RS due to retrogradation during physical, chemical, and enzymatic modifications. The formation of a double helix bond during retrogradation causes an increase in SDS and RS levels. Modification of HMT and MW had no significant effect (*p* > 0.05) in increasing VRSD and RDS (Fig. 5). VRDS levels of chemical, physical and enzymatic modification of starch have been used for retrogradation of breadfruit (22.10%) and corn (20%) starch (Barua *et al*. 2021).

The decrease in RDS with each modification came from changes in the amylose-amylopectin ratio. Black bean and pinto bean starch decreased RDS due to the short chain reassociation of amylose (Oh *et al*. 2022). Microwave heating of millet starch can change the starch features through gelatinisation, changing the molecular structure (Zailani *et al*. 2021). Lee *et al*. (2019) reported that the microwave cooking treatment for maize increased the RDS content by 30% (dw).

Modifications of AC-1C and AC-2C significantly increased (*p* < 0.05) the SDS content of corn flour when compared to control. Physical, chemical and enzymatic modifications significantly increased the levels of RS MCF (*p* < 0.05) (Fig. 5). The increase in SDS and RS levels in corn and rice was due to optimal retrogradation followed by the optimal formation of resistant starch (Lu *et al*. 2021). Research on barley flour showed that the characteristics of SDS and RS increased with the modification of AC-1C by 20.02% (dw) (Lee *et al*. 2019). Waxy corn flour contains a polyphenolic fraction, which significantly contributes to starch hydrolysis by inhibiting amylolytic enzymes (Oh *et al*. 2022). Annealing increases the SDS (20%) and RS (25%) content of corn starch through retrogradation and crystallisation (Larder *et al*. 2019). The original corn starch, without modification, had a lower RS content (2.05%) than starch that was sufficiently processed by cooking (8.18%) (Barua *et al*. 2021).

### Results of Analysis of MCF Resistant Starch

Modified annealing (ANN) increased the resistant starch content significantly (*p* < 0.05) by 39.52% when compared to control (K) by 4.42% with an increase of 8.94-fold (Table 1). Starch modified by the annealing process had lower amylose content (24.01%) and had lower RDS (16.02%) compared to control starch. Annealing treatment has been used to increase the content of SDS (20%) and RS (25%) in corn starch through retrogradation and crystallisation processes (Zavarezel *et al*. 2021).

Acid hydrolysis (HA) increased RS levels significantly (*p* < 0.05) by 23.98% when compared to the control which was 4.42%, with an increase of 5.43-fold (Table 1). The acid process hydrolyses starch granules increasing the RS and SDS content of MCF. There was an interaction relationship between acid and starch granules in corn flour which controlled amylase infiltration, this result was by the research of Li *et al*. (2018). Increasing the amount of cross-linked starch suppresses amylase infiltration through the porous starch channels, resulting in increased digestive resistance (Barua *et al*. 2021). The effect of crosslinking may contribute to the increase in RS content during starch and amylase synthesis (Chandrasekaran *et al*. 2013; Magallanes-Cruz *et al*. 2017; Sudheesh *et al*. 2020).

The debranching pullulanase modification gave a significant increase (*p* < 0.05) in increasing RS levels (17.16%) when compared to the control (K) which was 4.42% (Fig. 5) with an increase of 3.88-fold (Table 1). This is because the pullulanase enzyme breaks the α-1,6 glycosidic bond which is a branching bond in the amylopectin molecule (debranching) causing shortening of the amylose chain (DP 19–29) which is the raw material for RS (Ren *et al*. 2021).

### Resistance of MCF against Artificial Gastric Fluid *In Vitro*

Analysis of MCF resistance was tested against artificial gastric acid fluid at pH 2. The modification of corn flour by physical, chemical and enzymatic showed high resistance to gastric acid-simulated fluids (Table 2). Hydrolysis of gastric acid-simulated fluids is low because the MCF structure has undergone a complete retrogradation process to become resistant starch. Crystallisation occurs so that the double helix bond between amylose and amylopectin becomes stronger and solid, making it difficult to hydrolysis of gastric acid (Larder *et al*. 2019).

The increase in RS levels in corn starch, lotus and modified corn starch occurs due to starch’s chemical and physical modification, resulting in glycosidic bonds resistant to hydrolysis acetyl, ester and phosphate (Tu *et al*. 2021). Therefore, physical, chemical and enzymatic modification of corn flour will be an advantage for formulating low glycemic index foodstuffs specifically for people with *diabetes mellitus*.

### The Prebiotic Activity of MCF

Prebiotic activity is the ability of prebiotics to increase the growth of probiotic bacteria, which is associated with their selectivity to pathogenic bacteria (Putra 2020). Foodstuffs have a positive prebiotic activity if they are metabolised selectively by probiotic bacteria such as *L. acidophilus* and *L. plantarum* but not metabolised by pathogenic bacteria such as EPEC. On the other hand, food ingredients have a negative value of the prebiotic activity if the food is not selective so that EPEC pathogenic bacteria can metabolise it. The prebiotic properties (prebiotic activity, effect and index) were carried out on seven samples with each physical modification (HMT, annealing, autoclaving-cooling one cycle and two cycles, microwave), chemical modification with acid hydrolysis; enzymatic modification by debranching pullulanase. The probiotic lactic acid bacteria (LAB) tested, namely *Lactobacillus plantarum* IIA-1A5 has been claimed to be a probiotic bacterium that is beneficial for the human digestive system (Ji & Yu 2018; Morales-Medina *et al*. 2014). *L. plantarum* IIA-1A5 has microaerophilic to facultative anaerobic properties.

**Figure 6:**
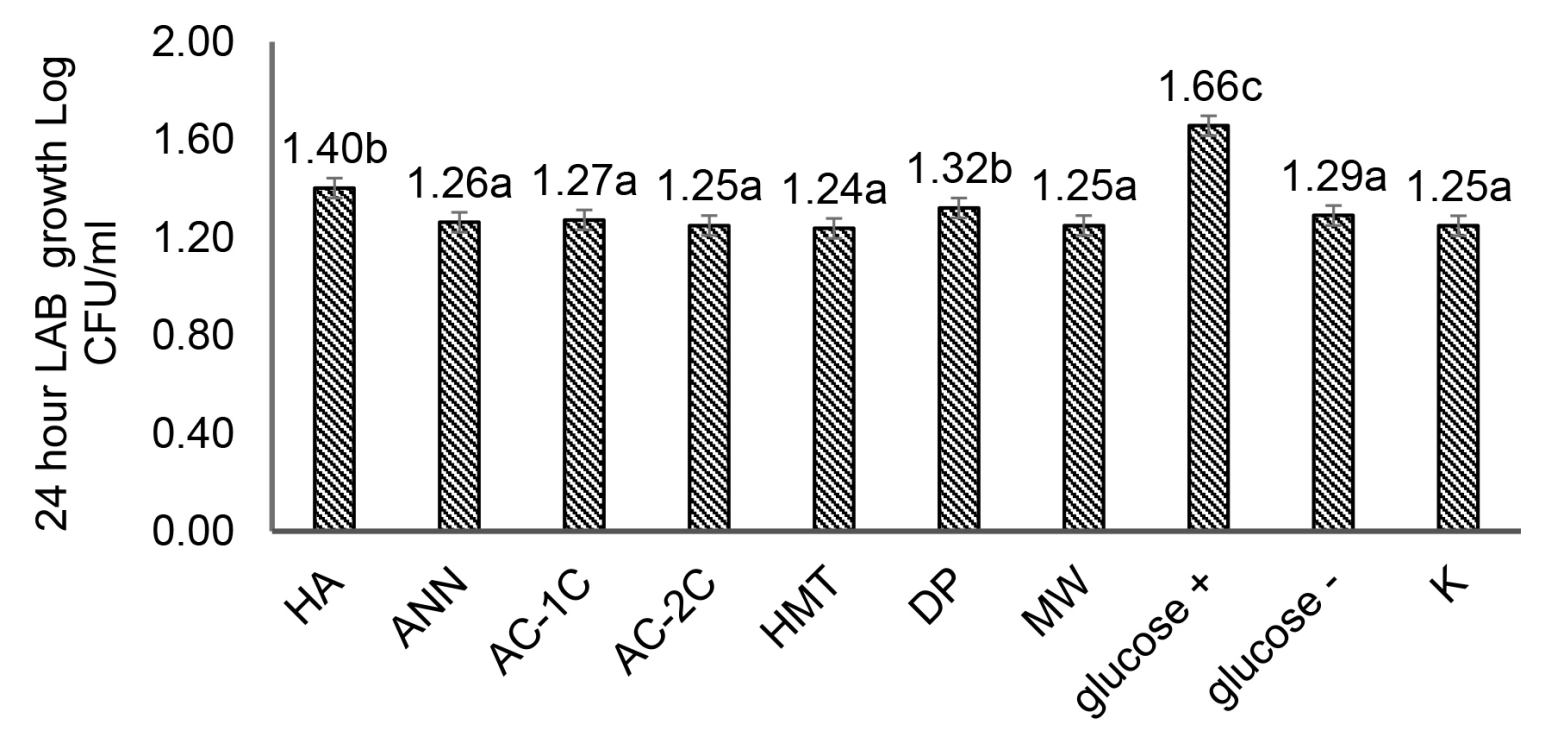

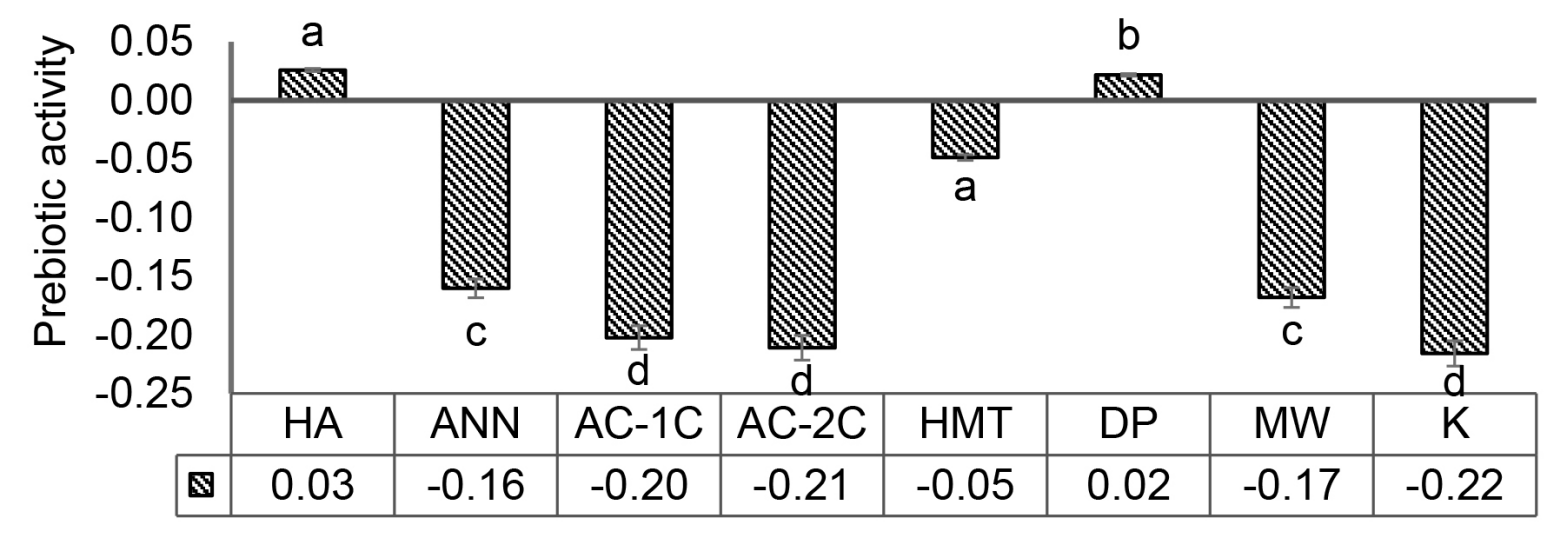
(a) Viability of *Lactobacillus plantarum* IIA-1A5; (b) Prebiotic activity of modified corn flour. HA (acid hydrolysis), ANN (annealing), AC-1C (1 cycle autoclaving-cooling), AC-2C (autoclaving-cooling 2 cycles), HMT (heat moisture treatment), DP (debranching pullulanase), MW (microwave) and K (control). The same letters in the bar chart show values that are not significantly different with a significance level of 95% (α = 5%), after statistical testing with Duncan on SPSS 26.

MCF was shown to have a significant effect (*p* < 0.05) on increasing the viability of *L. plantarum* IIA-1A5 through 24-hour incubation (Fig. 6). The viability of *L. plantarum* IIA-1A5 increased by 1.40 log CFU/mL on m-MRSB medium added with 2.5% MCF HA significantly (*p* < 0.05) when compared to control (1.25 log CFU/mL). The viability of *L. plantarum* IIA-1A5 increased by 1.32 log CFU/mL on a 2.5% MCF DP m-MRSB medium compared to the control (1.25 log CFU/mL) (Fig. 6). The prebiotic activity value of MCF HA was 0.03 with MCF DP 0.02; there was no significant difference (*p* > 0.05). Both MCF HA and MCF DP treatments are selective for pathogenic bacteria (EPEC), as shown in Fig. 6.

MCF acid hydrolysis (HA) and MCF debranching pullulanase (DP) showed positive values of 0.03 and 0.02, respectively, when tested as growth media for *L. plantarum* IIA-1A5. The MCF HA and MCF DP can be used as candidate sources of prebiotics because they have good effects, indexes and activities. MCF was used as a substitute for accessible carbon sources from reducing sugars so that only resistant starch, starch and oligosaccharides were available for growth. Research on purple sweet potato, rice and corn showed increased LAB growth with glucose as the primary nutrient (Janggu *et al*. 2021).

### Prebiotic Effect and Index of MCF

The prebiotic effect is an increase in the population of probiotic bacteria that occurs constantly without considering the concentration of prebiotics. While the prebiotic index is an increase in the population of probiotic bacteria associated with the concentration of prebiotics (Imelda *et al*. 2020; Putra 2020). Lactic acid bacteria (LAB), which have been widely used in the fermentation of foodstuffs such as *L. plantarum* SMN 25, *L. plantarum pentosus* SMN 01, and *L. plantarum pentosus* FNCC 235, isolated from traditional food fermentations, have shown the ability to produce galactosidase capable of degrading oligosaccharides (Ning *et al*. 2020). This study also evaluates the prebiotic properties of unfermented corn flour. Testing of prebiotic properties includes:

1. Resistance of the MCF to artificial gastric juices.
2. LAB viability, effect, and prebiotic index of MCF.
3. Prebiotic activity of MCF on the growth of pathogenic bacteria that cause diarrhea.

**Figure 7:**
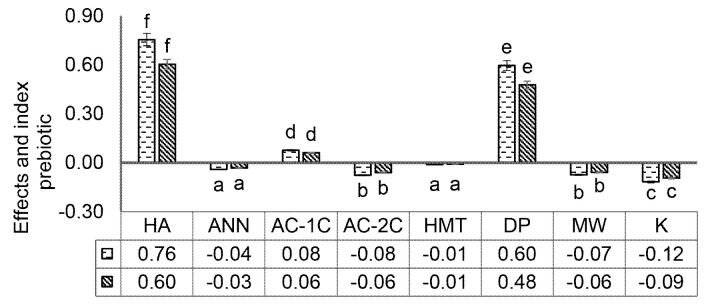
Prebiotic effects and prebiotic index of modified corn flour on *Lactobacillus plantarum* IIA-1A5. HA (acid hydrolysis), ANN (annealing), AC-1C (1 cycle autoclaving-cooling), AC-2C (autoclaving-cooling 2 cycles), HMT (heat moisture treatment), DP (debranching pullulanase), MW (microwave) and K (control). The same letters in the bar chart show values that are not significantly different with a significance level of 95% (α = 5%), after statistical testing with Duncan on SPSS 26.0.

The increase in the prebiotic effect of foodstuffs has a significant impact (*p* < 0.05) on the rise in the prebiotic index, as reported by Tu *et al*. (2021). The highest prebiotic effect of *L. plantarum* was shown in growth media containing MCF HA and DP (Fig. 7). The content of resistant starch contained in MCF can be used to increase the viability of probiotic LAB. The index value and the effect of prebiotics in this study did not reach 1. This value indicates that the type of growth media has lower effectiveness when compared to glucose of 2.14 in growing LAB as probiotic candidates, as has been reported in studies discussing sorghum and barley starch (Shaikh *et al*. 2019; Mapengo *et al*. 2021). Imelda *et al*. (2020) report that food is declared a good source of prebiotics if it has an effective value and a prebiotic index above 2.0 (Imelda *et al*. 2020). However, the results showed that MCF acid hydrolysis and debranching pullulanase could stimulate the viability of probiotic LAB, so they were still influential in being developed as prebiotic candidates.

## CONCLUSION

Modified corn flour showed very high hydrolysis resistance, more than 95% against artificial gastric acid. The prebiotic activity value of MCF showed modified Modified HA of 0.03 and a debranching pullulanase (DP) of 0.02. The value of the prebiotic effect of MCF HA (0.76) and DP (0.60). Prebiotic index value of HA (0.60) and DP (0.48). MCF HA and DP are good prebiotic candidates because having high levels of resistant starch (39.52%; 17.16%), low starch digestibility (82.35%; 70.32%), and artificial gastric acid hydrolysis (100%; 96.83%). MCF HA and DP had better prebiotic activity, effect and index than control corn flour without modification.

## Figures and Tables

**Table 1:** Increased levels of resistant starch (RS) of Momala corn flour.

Sample	Average RS level (%)	Increased level of RS (fold)
HA	23.98	5.4
ANN	39.52	8.9
AC-1C	13.18	2.9
AC-2C	9.00	2.0
HMT	4.73	1.0
DP	17.16	3.8
MW	6.04	1.3
K	4.42	0.0

*Notes:* RS improvement is the difference between modified RS and control RS. HA = acid hydrolysis, ANN = annealing, AC-1C = autoclaving-cooling 1 cycle, AC-2C = autoclaving-cooling 2 cycles, HMT = heat moisture treatment, DP = debranching pullulanase, MW = microwave-cooling, K = control

**Table 2:** Results of modified corn flour resistance to gastric acid hydrolysis.

Sample	MCF resistance to hydrolysis (%)					

	Incubation time (hours)					

	0	0.5	1	2	4	6
HA	100 ± 0	97.12 ± 0.02	94.95 ± 0.06	93.18 ± 0.04	91.15 ± 0.07	89.72 ± 0.03
ANN	100 ± 0	97.85 ± 0.07	91.44 ± 0.05	87.21 ± 0.02	83.89 ± 0.08	81.54 ± 0.08
AC-1C	100 ± 0	96.12 ± 0.08	93.29 ± 0.05	90.12 ± 0.06	86.47 ± 0.09	85.08 ± 0.02
AC-2C	100 ± 0	97.34 ± 0.01	94.39 ± 0.03	91.64 ± 0.09	88.45 ± 0.07	86.23 ± 0.02
HMT	100 ± 0	96.87 ± 0.04	92.55 ± 0.04	88.15 ± 0.05	85.20 ± 0.08	81.88 ± 0.08
DP	100 ± 0	97.58 ± 0.05	95.12 ± 0.07	93.67 ± 0.05	91.65 ± 0.01	90.88 ± 0.07
MW	100 ± 0	98.34 ± 0.04	92.17 ± 0.07	89.91 ± 0.07	85.78 ± 0.02	83.18 ± 0.03
K	100 ± 0	96.72 ± 0.03	90.87 ± 0.07	85.19 ± 0.06	80.13 ± 0.06	74.28 ± 0.05

*Notes:* HA = acid hydrolysis, ANN = annealing, AC-1C = autoclaving-cooling 1 cycle, AC-2C = autoclaving-cooling 2 cycles, HMT = heat moisture treatment, DP = debranching pullulanase, MW = microwave, and K = control
